# Causal validation of the relationship between 35 blood and urine biomarkers and hyperthyroidism: a bidirectional Mendelian randomization study and meta-analysis

**DOI:** 10.3389/fendo.2024.1430798

**Published:** 2024-08-12

**Authors:** Wanxian Xu, Jiao Wu, Daolei Chen, Rui Zhang, Yue Yang

**Affiliations:** Department of Breast and Thyroid Surgery, First People’s Hospital of Kunming City & Calmette Affiliated Hospital of Kunming Medical University, Kunming, Yunnan, China

**Keywords:** blood and urine biomarkers, hyperthyroidism, Mendelian randomization analysis, meta-analysis, authenticate reverse, multiple corrections

## Abstract

**Background:**

Hyperthyroidism is an endocrine disorder with a relatively low global prevalence but significantly higher incidence among females compared to males. The onset age primarily ranges from 30 to 50, although it is not limited to this age group. Challenges in the treatment of hyperthyroidism include individualized treatment plan formulation, management of side effects, and prediction of disease progression, necessitating comprehensive consideration to achieve more effective therapy and management. Mendelian randomization studies can reveal more precise therapeutic targets between blood and urine biomarkers and hyperthyroidism, providing more decadent treatment options for the condition.

**Methods:**

The study will build upon the omics Mendelian randomization (MR) framework by conducting MR analysis using 35 blood and urine biomarkers separately for two distinct databases of hyperthyroidism. Subsequently, the results will undergo meta-analysis and multiple corrections to ensure accuracy and reliability. Finally, positive findings will undergo reverse MR validation to verify causal relationships with hyperthyroidism.

**Results:**

In the British database, the MR analysis of Total bilirubin levels about hyperthyroidism yielded an odds ratio (*OR*) of 1.097 (95% *CI*: 0.951-1.265, *P* = 0.205). Conversely, in the Thyroid Omics Association database, the MR analysis revealed an *OR* of 1.283 (95% *CI*: 1.122-1.467, *P* = 0.0002) for the same relationship. Meta-analysis of the MR analysis results from both databases, following multiple corrections, resulted in an *OR* of 1.192 (95% *CI*: 1.081-1.314, *P* = 0.015). Additionally, the direction of beta values in the MR analysis results from both databases was consistent.

**Conclusion:**

The urine biomarker total bilirubin levels may contribute to an increased risk of hyperthyroidism and accelerate its progression, thus representing a risk factor for the condition.

## 1 Introduction

Hyperthyroidism is a condition caused by the overactivity of the thyroid gland, manifesting as symptoms of hypermetabolism such as increased body temperature, elevated heart rate, and weight loss, often accompanied by anxiety and cardiovascular symptoms like palpitations. About one-third of patients experience ocular symptoms such as protrusion of the eyeballs and eyelid swelling. Additionally, hyperthyroidism can lead to thyroid enlargement, muscle weakness, irregular menstruation, and other issues (1–3). Timely diagnosis and treatment are crucial for preventing complications.

Hyperthyroidism is an endocrine disorder with a global prevalence of approximately 0.5% to 2%, with females being 5 to 10 times more likely to be affected than males. While the most common onset age is between 30 and 50 years old, hyperthyroidism can occur at any age. Environmental, geographical, and genetic factors influence regional variations in prevalence. Family history is one of the significant risk factors for developing hyperthyroidism, with individuals having a family history being at higher risk (4, 5).

Treatment of hyperthyroidism faces several significant challenges. Firstly, formulating personalized treatment plans is crucial as patients’ treatment needs vary due to individual differences, requiring the development of the best plan tailored to each patient’s condition and lifestyle. Secondly, conventional treatments such as antithyroid drugs, radioactive iodine therapy, and thyroidectomy may be associated with some degree of side effects, such as drug allergies and hypothyroidism, which require effective management. Additionally, predicting the progression of hyperthyroidism is complex, with some patients experiencing recurrence or transitioning to hypothyroidism, increasing the need for long-term patient follow-up and management. Therefore, in the treatment of hyperthyroidism, challenges such as personalized treatment, side effect management, and disease progression prediction need to be comprehensively considered to achieve more effective treatment and management (6–10).

In the diagnosis and monitoring of hyperthyroidism, blood and urine biomarkers play a significant role, involving a series of pathophysiological mechanisms. Common blood and urine biomarkers include thyroid-stimulating hormone (TSH), which is usually decreased because high levels of thyroid hormones (T3 and T4) inhibit the secretion of TSH from the pituitary gland via a negative feedback mechanism. Elevated total triiodothyronine (total T3) and free triiodothyronine (free T3) levels are typical features of hyperthyroidism, with the increase in free T3 being more pronounced since it is not restricted by plasma protein binding and directly reflects the biological activity of thyroid hormones. Total thyroxine (total T4) and free thyroxine (free T4) levels are usually also elevated, with free T4 representing the actual biological activity (11). In Graves’ disease, the most common cause of hyperthyroidism, thyroid receptor antibody (TRAb) levels are significantly elevated. These antibodies bind to TSH receptors on thyroid cells, mimicking the action of TSH and continuously stimulating the thyroid to secrete excess T3 and T4. Additionally, anti-thyroid peroxidase antibodies (TPOAb) and anti-thyroglobulin antibodies (TgAb) are usually elevated in autoimmune thyroid diseases, reflecting the immune system’s attack on the thyroid (12).

Hyperthyroidism also leads to an accelerated metabolism, affecting electrolyte balance and blood glucose levels. High levels of thyroid hormones can increase intestinal absorption and renal excretion of potassium, leading to hypokalemia, and accelerate glucose metabolism, leading to elevated blood glucose levels. Furthermore, total bilirubin levels may also be affected by hyperthyroidism, with the specific pathophysiological mechanisms including: 1. Impact of Elevated Bilirubin on Thyroid Hormone Metabolism: The liver plays a key role in the metabolism of thyroid hormones, particularly the conversion of T4 to T3. Elevated total bilirubin levels often indicate liver dysfunction, which can hinder thyroid hormone metabolism, disrupting the balance of T3 and T4 in the serum and potentially triggering hyperthyroidism. Specifically, liver dysfunction may reduce the activity of deiodinase enzymes responsible for converting T4 to the more active T3, leading to an accumulation of T4 and a deficiency of T3. However, under certain conditions, the accumulation of T4 may increase T3 production through other pathways, ultimately causing an imbalance in thyroid hormone levels. Additionally, bilirubin and thyroid hormones both need to bind to plasma proteins for transport in the blood. Elevated bilirubin levels may competitively inhibit the binding of thyroid hormones to plasma proteins, increasing the concentration of free thyroid hormones and further contributing to hyperthyroidism. The increase in free thyroid hormones directly affects the feedback regulation mechanism of the thyroid, causing the thyroid to secrete more hormones and forming a vicious cycle. 2. Activation of the Immune System and Inflammatory Response: Elevated total bilirubin levels may be associated with inflammation of the liver or biliary system. These inflammatory responses can affect thyroid function through systemic inflammation. Immune responses induced by liver inflammation may attack thyroid tissue, triggering hyperthyroidism. Elevated levels of inflammatory factors such as TNF-α and IL-6 can directly damage liver cells and affect thyroid cell function through immune-mediated mechanisms. Furthermore, immune system activation caused by elevated bilirubin may exacerbate or trigger autoimmune hyperthyroidism, such as Graves’ disease. Graves’ disease is the most common cause of hyperthyroidism and is essentially an autoimmune disease where the immune system attacks thyroid tissue, leading to excessive secretion of thyroid hormones. In Graves’ disease patients, TRAb levels are usually elevated, stimulating the thyroid and causing excessive hormone secretion. Systemic immune activation triggered by elevated bilirubin may be one of the factors inducing TRAb production, further promoting the development of hyperthyroidism. 3. Metabolic Disorders in the Body: Elevated bilirubin levels are often accompanied by increased oxidative stress. Oxidative stress can impair the normal function of cells, including thyroid cells, affecting the secretion and metabolism of thyroid hormones and leading to hyperthyroidism. Oxidative stress can cause lipid peroxidation of cell membranes, DNA damage, and oxidative modification of proteins, thereby affecting the normal physiological functions of cells. Additionally, liver dysfunction and elevated bilirubin levels can affect the metabolism and regulation of various hormones in the body, leading to an overall imbalance in the endocrine system, including the regulation of thyroid hormones. Specifically, impaired liver metabolism of thyroid hormones may lead to a decrease in hormone clearance rates and an increase in serum thyroid hormone levels. When total bilirubin levels are elevated, the metabolic load on the liver increases, which is not limited to bilirubin but also includes other metabolic products. Overload of liver function may lead to the accumulation of metabolic products, including thyroid hormones, causing hyperthyroidism. Metabolic disorders may also affect the expression and function of thyroid hormone receptors, causing thyroid feedback regulation to fail and further exacerbating the development of hyperthyroidism (13–15).

Although there is currently limited direct research exploring the causal relationship between blood and urine biomarkers and hyperthyroidism, these mechanisms provide a theoretical basis for exploring their potential links. This study investigates the causal relationship between specific blood and urine biomarkers and hyperthyroidism through Mendelian randomization, revealing clearer pathophysiological mechanisms between the two and providing new ideas for clinical diagnosis and treatment.

MR studies based on genome-wide association study (GWAS) data are a method of causal inference using GWAS data. By identifying genetic variants (SNPs) associated with the trait of interest and using these SNPs as instrumental variables, randomized experiments can be simulated to assess the causal effects of genes on specific traits. The causal relationship between specific genes and traits can be determined through statistical analysis rather than just correlation. Although challenges such as SNP selection and consideration of confounding variables exist, this approach provides essential clues for understanding disease occurrence and treatment (16, 17). It helps reveal the effects of genes on complex traits.

Currently, MR studies of hyperthyroidism have covered various aspects, but the causal relationship between blood and urine biomarkers and hyperthyroidism has yet to be thoroughly explored. To fill this research gap, we plan to conduct an MR study based on two samples, focusing on the causal relationship between 35 omics blood and urine biomarkers and hyperthyroidism. Through this study, we aim to uncover the potential links between blood and urine biomarkers and the risk of hyperthyroidism, providing new insights and clues for the early diagnosis and treatment of hyperthyroidism.

## 2 Materials and methods

### 2.1 Study design

**Figure 1:**
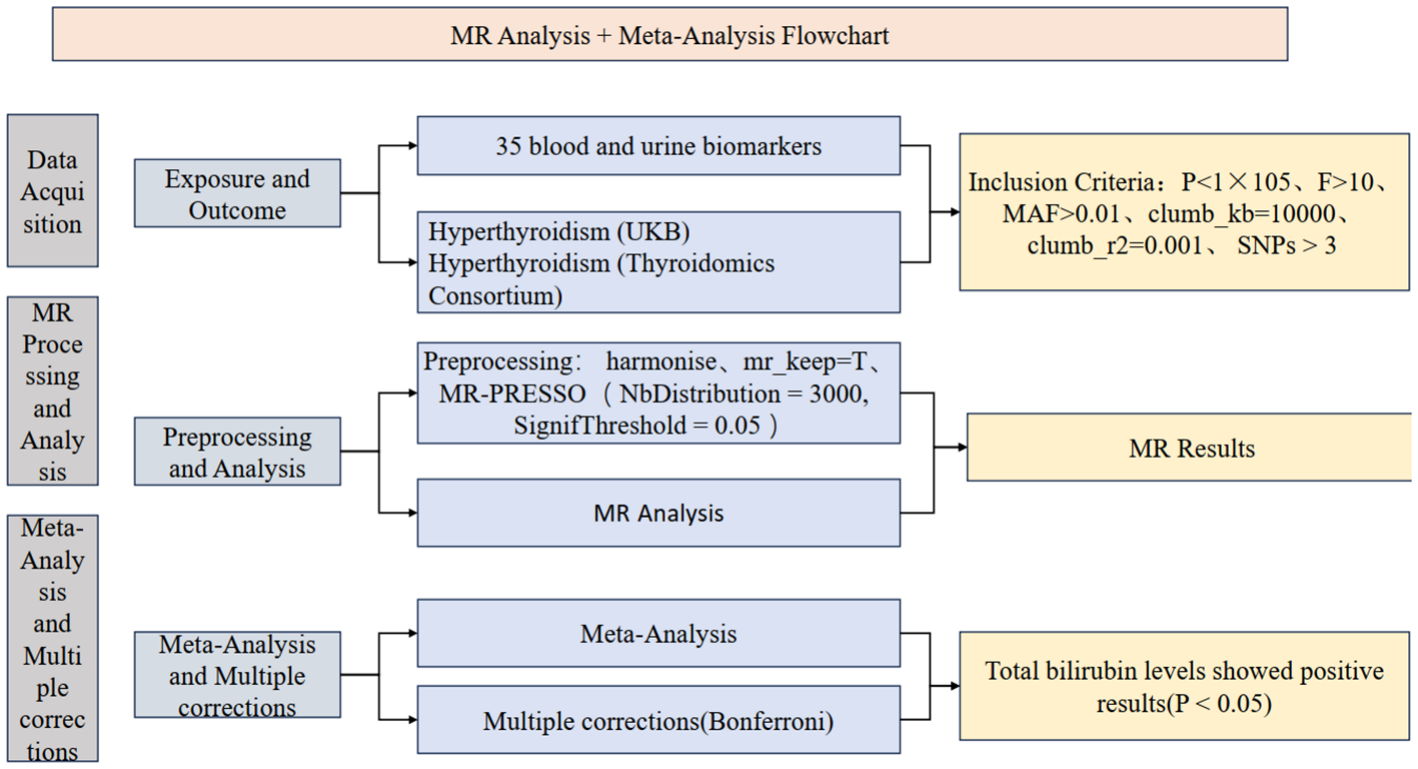
The process flowchart of the research methodology.

The study is primarily divided into three parts: firstly, obtaining exposure and outcome data for the research, followed by preprocessing and selection of instrumental variables; secondly, conducting Mendelian randomization (MR) analysis and sensitivity analysis for the 35 blood and urine biomarkers separately with hyperthyroidism in two databases, along with visualization; finally, a meta-analysis of the main results from the analysis, integrating results from different databases, and conducting multiple corrections to ensure the robustness of the results. The study also includes the creation of a flowchart to demonstrate the research process. Relevant institutions and committees have approved all data involved, and participants have provided informed consent. Flowcharts have been created (**Figure 1**), and the STROBE-MR checklist (18) has been completed to better understand the study.

### 2.2 The source of GWAS data for 35 types of hematuria

The study utilized data from the GWAS Catalog, analyzing samples from 341,077 individuals of European descent. The genetic characteristics of 35 blood and urine biomarkers were investigated, revealing the genetic basis of these biomarkers. Through genome-wide association studies (GWAS), multiple genetic loci significantly associated with these biomarkers were identified, showing that most biomarkers have a significant genetic component. Specifically, blood biomarkers such as glucose, cholesterol, bilirubin, and inflammatory markers, as well as urine biomarkers such as uric acid and urinary protein, demonstrated various genetic associations and related genes. These findings contribute to understanding the impact of genetics on biomarker levels, aiding in disease risk prediction and providing a foundation for personalized medicine and precision medicine research. Specifically, the GWAS IDs for the 35 blood and urine biomarkers studied range from GCST90019492 to GCST90019526 (19).

Based on recent research, we applied the following criteria for screening: P < 1×10^-5^, F > 10, MAF > 0.01, clump_kb = 10000, and clump_r2 = 0.001. The final number of SNPs meeting the criteria was 17,981.

### 2.3 Source of the dataset for hyperthyroidism

The outcome datasets included in this study consist of two groups from two different databases, both distinct from the exposure data sources to avoid data overlap. Specifically, the first group of hyperthyroidism data comes from the UK database (download link: [https://pan-ukb-us-east-1.s3.amazonaws.com/sumstats_flat_files/categorical-20002-both_sexes-1225.tsv.bgz]), with 3,197 cases, 417,276 controls, and 13,984,704 SNPs (Pan-UKB team. https://pan.ukbb.broadinstitute.org. 2020.). The baseline characteristics of the patients include data from the UK Biobank, covering genotypic data and electronic health records of approximately 500,000 individuals, as well as survey data. Participants’ ages range from 40 to 69 years, primarily focusing on the prevention, diagnosis, and treatment of late-onset severe diseases such as diabetes, cancer, arthritis, heart disease, stroke, and dementia. The study population consists entirely of individuals of European ancestry.

The second dataset comes from the Thyroidomics Consortium (download link: [https://transfer.sysepi.medizin.uni-greifswald.de/gwasresults/formatted_decreasedTSH_overall_130421_invvar1.txt-QCfiltered_GC.txt.gz]), with 1,840 cases, 51,823 controls, and 4,370,606 SNPs (20). Baseline characteristics include participants of European descent, involving meta-analyses of genome-wide association studies (GWAS) of over 50,000 individuals. These analyses cover reference ranges for thyroid-stimulating hormone (TSH), free thyroxine (FT4), free and total triiodothyronine (T3), and metabolic markers (T3/FT4 ratio). The study also analyzed dichotomous data on high and low TSH levels.

Inclusion and exclusion criteria for the study data:

#### 2.3.1 Poor data quality control

SNP data that fail quality control (QC) checks, such as high missing rates or low minor allele frequency (MAF), and datasets that have not undergone stringent QC processes, such as the removal of SNPs or samples with high missing rates, should be excluded. The data in this study are complete and reliable.

#### 2.3.2 Population structure issues

Datasets with significant population stratification that has not been effectively corrected, and individuals with inconsistent ancestral backgrounds within the dataset, should be excluded to avoid population admixture. In this study, all data populations are of European ancestry.

#### 2.3.3 Sample overlap

Ensure there is no sample overlap between exposure data and outcome data. Duplicates or closely related individuals (e.g., family members) within the dataset should be excluded. The exposure data and the two groups of outcome data in this study are sourced from different databases, minimizing the risk of cross-data overlap.

#### 2.3.4 Insufficient statistical power

Studies with a sample size too small to detect the expected effects, leading to low statistical power, should be excluded. The data used in this study are from datasets with the optimal sample size available in the respective databases.

#### 2.3.5 Data source and reliability issues

Data from unreliable sources, collected using non-rigorous methods, or lacking detailed descriptions and documentation support, as well as datasets with insufficient openness and transparency, should be excluded. The data in this study are sourced from publicly available databases.

### 2.4 Criteria for selection of instrumental variables

In conducting MR studies, the selection of instrumental variables is crucial. According to recent research, the threshold for P-values is typically set at 1×10^-5^ to ensure an adequate number of SNPs are included in the study (17). Subsequently, the F-statistic is calculated based on known variables using the formula F = (beta/se)^2^ (**Supplementary Table 1**). Only SNPs with an F-value greater than 10 are retained, helping to eliminate weak instrumental variables and thereby enhancing the reliability of the study results (21, 22).

Additionally, further SNP filtering is necessary to minimize the impact of rare variants on the study results. The minor allele frequency (MAF) is calculated based on the effect allele frequency (eaf), where MAF is set to eaf if eaf is less than 0.5, otherwise it is set to 1-eaf. Only SNPs with an MAF greater than 0.01 are retained to eliminate interference from rare variants on the study results (23, 24).

Finally, the filtered data is formatted into MR format, and linkage disequilibrium (LD) processing is applied to ensure the independence of instrumental variables and the accuracy of the results. LD processing criteria include setting the distance threshold to 10,000 kilobase pairs (kb) and the LD threshold to 0.001 (25, 26). The execution of these steps ensures the reliability and accuracy of MR studies, laying a solid foundation for further data analysis.

## 3 Statistical analysis

### 3.1 Validation of causality between 35 hematuria biomarkers and hyperthyroidism

All data analysis in this study was conducted using R version 4.2.1 (https://www.r-project.org/). Firstly, we selected SNPs related to hyperthyroidism from outcome databases that differ from the exposure data source. We matched them with SNPs in the exposure data, retaining only matched data. Next, based on the specific criterion action = 2, we processed palindrome SNPs in the data. Additionally, data with mr_keep = false were excluded (27).

Before conducting MR-PRESSO processing, we performed a horizontal pleiotropy test on the processed data. If the p-value of an SNP is less than 0.05, we identify it as horizontally pleiotropic positive, indicating outliers. Subsequently, we use the MR-PRESSO method to remove these outliers and ensure data accuracy. The specific parameters for MR-PRESSO are NbDistribution=3000 and SignifThreshold=0.05. For SNPs with p-values greater than 0.05, we consider them to have no outliers (28, 29).

After fine-tuning the data, before conducting MR analysis, we performed a heterogeneity test on the data. Although data heterogeneity has a minor impact on the results, for optimization, we use the IVW random-effects model for MR analysis of SNPs with heterogeneity (Q_pval < 0.05); for SNPs with insignificant heterogeneity, we use the IVW fixed-effects model. Additionally, regardless of heterogeneity, we conducted MR-Egger, weighted median methods analysis on the data, and calculated the OR (30, 31) (**Supplementary Table 2**). To enhance result reliability, we conducted a meta-analysis of the analysis results of blood and urine biomarkers with hyperthyroidism from two databases, specifically a meta-analysis of IVW in MR results (32, 33) (**Supplementary Table 3**). After analysis, multiple corrections were applied to the meta-analysis results to reduce the possibility of type I errors, using the Bonferroni method for multiple corrections. Ultimately, only one set of blood and urine biomarkers showed positive results after meta-analysis and multiple corrections (**Supplementary Table 4**).

### 3.2 Causal link between hyperthyroidism and the positive hematuria marker

To investigate the potential bidirectional relationship between the final positive blood and urine biomarkers and hyperthyroidism, the final positive blood and urine biomarkers are treated as outcome data, while hyperthyroidism is considered exposure data. The instrumental variable selection and data analysis process remains consistent with the forward analysis.

This process primarily aims to validate the directionality of the causal relationship between the two. By analyzing hyperthyroidism as the exposure and the identified blood and urine biomarkers as outcomes (**Supplementary Table 5**), we can assess whether the relationship observed in the forward analysis holds in the reverse direction (34, 35). This approach helps to elucidate whether the identified biomarkers contribute to the development of hyperthyroidism or vice versa.

### 3.3 Sensitivity analysis

Horizontal pleiotropy is when different treatment methods or interventions may affect individuals or circumstances. These effects may be mistakenly attributed to differences between the experimental and control groups rather than the actual treatment effect. To mitigate the impact of horizontal pleiotropy on experimental results, we conducted a horizontal pleiotropy test on the GWAS data. For SNPs exhibiting horizontal pleiotropy (pval < 0.05), we used MRPRESSO to remove outliers from the data, with specific exclusion criteria set as NbDistribution=3000 and SignifThreshold=0.05 (**Supplementary Table 6**).

Heterogeneity refers to diversity or differences in study subjects, observations, or experimental conditions. In statistics and research methodology, heterogeneity typically refers to differences between samples or individuals, which may stem from individual characteristics, environmental conditions, or other factors. Heterogeneity is common in research and can manifest in various aspects, including physiological and psychological differences between individuals, socioeconomic status, and the influence of environmental factors, among others. This diversity and variability contribute to the generalizability and representativeness of research results but also increase the complexity and difficulty of interpretation.

We similarly conducted a heterogeneity test on the data during the analysis process. For SNPs exhibiting heterogeneity (Q_pval < 0.05), we used a random-effects model in the IVW MR analysis process to ensure the accuracy and reliability of the results. Otherwise, we employed a fixed-effects model (**Supplementary Table 7**).

## 4 Results

### 4.1 Validation of causality between 35 hematuria biomarkers and hyperthyroidism

**Figure 2:**
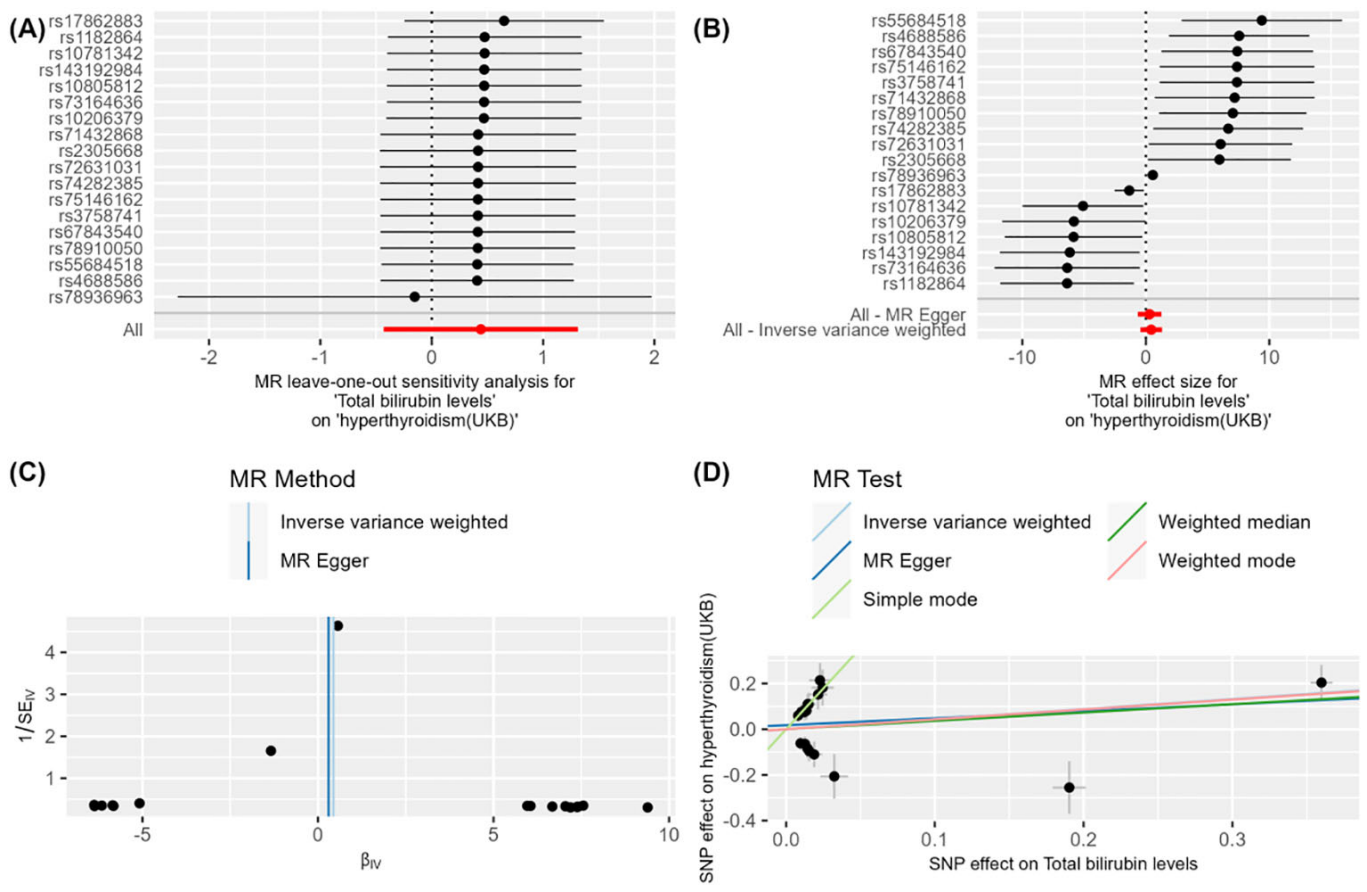
**Figure 2** demonstrates the impact of total bilirubin levels on hyperthyroidism (UKB) using Mendelian Randomization (MR) analysis. **(A)** shows a leave-one-out sensitivity analysis, indicating that the overall effect remains robust when each SNP is removed one at a time, suggesting that individual SNPs have minimal impact on the overall effect. **(B)** presents a forest plot showing the effect size and confidence intervals of each SNP on the impact of "total bilirubin levels" on "hyperthyroidism (UKB)." The combined results calculated using "MR Egger" and "Inverse Variance Weighted" methods indicate a significant effect. **(C)** is a funnel plot depicting the relationship between SNP effect sizes and standard errors to detect publication bias. The points are evenly and symmetrically distributed, indicating no apparent publication bias. **(D)** shows a scatter plot where different MR methods (Inverse Variance Weighted, MR Egger, Weighted Median, and Weighted Mode) exhibit consistent β values, indicating a causal effect of total bilirubin levels on hyperthyroidism (UKB).

**Figure 3:**
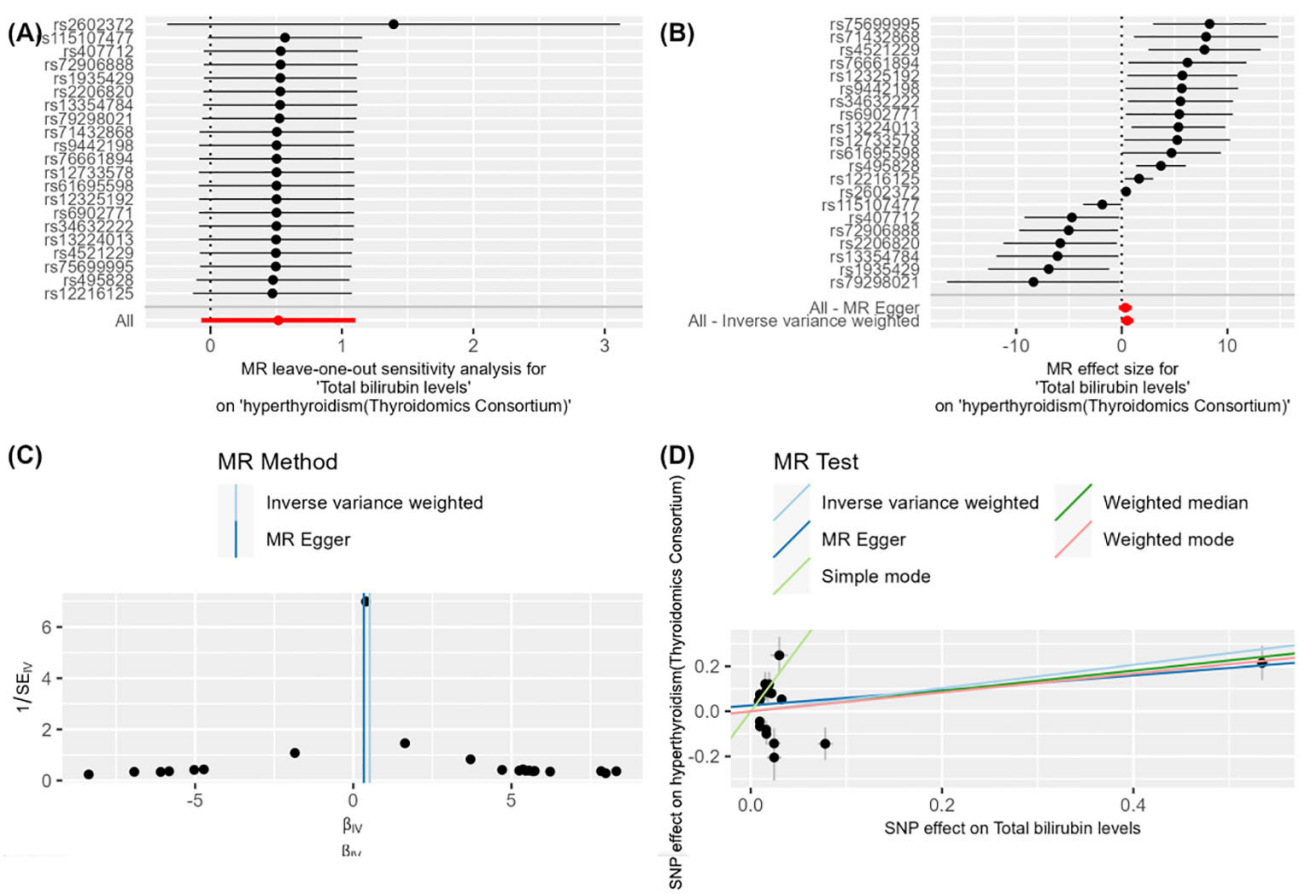
**Figure 3** demonstrates the impact of total bilirubin levels on hyperthyroidism (Thyroidomics Consortium) using Mendelian Randomization (MR) analysis. **(A)** shows a leave-one-out sensitivity analysis, indicating that the overall effect remains robust when each SNP is removed one at a time, suggesting that individual SNPs have minimal impact on the overall effect. **(B)** presents a forest plot showing the effect size and confidence intervals of each SNP on the impact of "total bilirubin levels" on "hyperthyroidism (Thyroidomics Consortium)." The combined results calculated using "MR Egger" and "Inverse Variance Weighted" methods indicate a significant effect. **(C)** is a funnel plot depicting the relationship between SNP effect sizes and standard errors to detect publication bias. The points are evenly and symmetrically distributed, indicating no apparent publication bias. **(D)** shows a scatter plot where different MR methods (Inverse Variance Weighted, MR Egger, Weighted Median, and Weighted Mode) exhibit consistent β values, indicating a causal effect of total bilirubin levels on hyperthyroidism (Thyroidomics Consortium).

**Figure 4:**
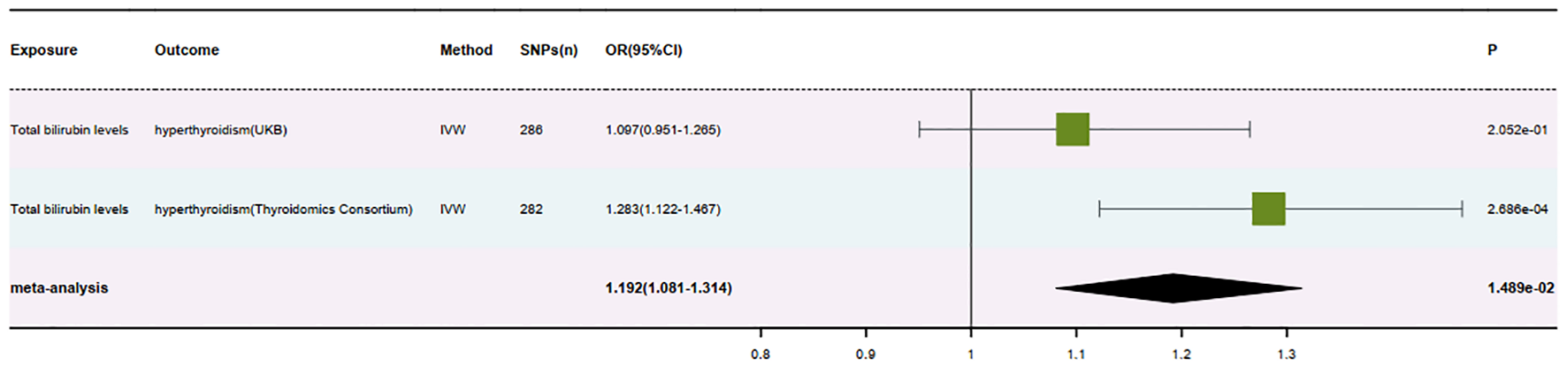
Forest plot of positive results after meta-analysis.

After meticulous analysis and multiple corrections, we found that only one group of blood biomarkers is causally associated with hyperthyroidism. Specifically, this blood biomarker is Total bilirubin levels (GCST90019521). In the UK database, MR analysis of hyperthyroidism revealed an *OR* value of 1.097 (95%*CI*: 0.951-1.265, *P* = 0.205), a combined graph of the MR results was plotted (**Figure 2**). In the Thyroid Omics Association database, the MR analysis showed an OR value of 1.283 (95%*CI*: 1.122-1.467, *P* = 0.0002) (**Supplementary Table 2**), and a combined graph of the MR results was plotted (**Figure 3**). Subsequently, we conducted a meta-analysis of the MR analysis results of hyperthyroidism in these two databases (**Supplementary Table 3**), and performed multiple corrections, resulting in an *OR* value of 1.192 (95%*CI*: 1.081-1.314, *P* = 0.015) (**Supplementary Table 4**). We also plotted a forest plot for the meta-analysis and the corrected results (**Figure 4**). Notably, the direction of beta values in the MR analysis results of this blood biomarker in both databases is consistent. Overall, these findings suggest that Total bilirubin levels are a risk factor for hyperthyroidism, potentially exacerbating the progression of this condition.

### 4.2 Causal link between hyperthyroidism and the positive hematuria marker

When conducting MR analysis with the positive blood biomarker as the outcome and hyperthyroidism in the UK database as the exposure, the *OR* value was 1.011 (95%*CI*: 0.999-1.023, *P* = 0.148). However, when hyperthyroidism from the Thyroid Omics Association was considered the exposure, the results showed an *OR* value of 0.989 (95%*CI*: 0.981-0.998, *P* = 0.043). Although the results from the Thyroid Omics Association database were statistically significant (*P* = 0.043), the direction of beta values across various methods in the MR analysis did not align consistently, precluding it from being established as a positive result. These findings indicate that the blood biomarker Total bilirubin levels only unidirectionally influences hyperthyroidism, and there is no evidence of hyperthyroidism affecting Total bilirubin levels in return (**Supplementary Table 5**). This process elaborates more precisely on the directionality between the two.

## 5 Discussion

Elevated total bilirubin levels are an essential biochemical indicator to assess liver function and biliary tract health. Bilirubin is a yellow pigment produced from the breakdown of red blood cells, which undergoes various metabolic processes in the body before being excreted by the liver (36, 37). Total bilirubin levels typically include both direct and indirect bilirubin components.

Direct Bilirubin: Also known as conjugated bilirubin, it is formed when the liver converts indirect bilirubin into a water-soluble form. Elevated direct bilirubin levels may indicate liver dysfunction or issues such as biliary obstruction (38, 39). Indirect Bilirubin: Also known as unconjugated bilirubin, it is produced from the breakdown of red blood cells and is converted into direct bilirubin inside the liver. Elevated indirect bilirubin levels may result from excessive breakdown of red blood cells, liver dysfunction, or biliary obstruction (40, 41). Measurement of total bilirubin levels is typically conducted through blood tests. Typical ranges vary depending on laboratory methods and standards, but normal total bilirubin levels in adults generally range from 0.3 to 1.9 milligrams per deciliter (42).

In recent years, research on the relationship between human metabolites and Graves’ disease has been increasing. Metabolomics studies have shown that Graves’ disease patients exhibit significant differences in amino acid, lipid, and carbohydrate metabolism compared to healthy individuals. For example, levels of glutamic acid and tyrosine in the serum of patients are elevated, cholesterol and triglycerides show abnormal levels, and there are changes in glucose metabolism products. These changes reflect metabolic disorders associated with the disease. Additionally, some metabolites are considered potential biomarkers for Graves’ disease, aiding in early diagnosis and prognosis assessment. Research also indicates that elevated oxidative stress metabolites are related to the immune response in Graves’ disease, and antioxidant treatment may benefit patients. Future research should further explore the specific mechanisms of these metabolites in Graves’ disease and combine genomics, transcriptomics, and other multi-omics technologies to develop more effective diagnostic tools and treatments, thereby improving the quality of life for patients (43, 44). A recent study used atmospheric ionization mass spectrometry to analyze carbonyl metabolic fingerprints in the urine of Graves’ disease patients, finding that the concentration of multiple metabolites was significantly higher in these patients compared to healthy controls. Several specific metabolic biomarkers were identified. These findings are crucial for understanding the metabolic mechanisms of Graves’ disease and exploring the potential application of these metabolic biomarkers in early diagnosis and personalized treatment. The study suggests that urine-based metabolite detection can serve as a non-invasive diagnostic and monitoring method for Graves’ disease, providing new possibilities for future clinical applications (45). Building on this theory, our research employs Mendelian randomization combined with meta-analysis to investigate the causal association of specific blood and urine biomarkers with hyperthyroid diseases.

In existing research, there are diverse studies on the relationship between bilirubin and diseases. Bilirubin has been studied as a protective factor in various diseases: 1. Bilirubin and Cardiovascular Diseases: Multiple studies have shown that high bilirubin levels have antioxidant and anti-inflammatory effects, which can reduce the risk of cardiovascular diseases. Bilirubin, as an endogenous antioxidant, directly scavenges free radicals and inhibits lipid peroxidation, reducing oxidative stress-induced cellular damage. It has been found that bilirubin can inhibit the oxidation of low-density lipoprotein (LDL), a major pathogenic factor in atherosclerosis. By reducing LDL oxidation, bilirubin can slow the progression of atherosclerosis and, consequently, lower the risk of cardiovascular diseases. Additionally, bilirubin can inhibit inflammatory responses by suppressing the release of various inflammatory mediators (e.g., TNF-α, IL-6), thus reducing inflammation-induced damage to vascular endothelial cells and preventing atherosclerosis and other cardiovascular diseases. Large-scale epidemiological studies have found that individuals with higher serum bilirubin levels have a significantly lower incidence of cardiovascular diseases, further supporting the potential protective role of bilirubin in cardiovascular health. 2. Bilirubin and Metabolic Syndrome: Bilirubin has been found to be inversely associated with components of metabolic syndrome, such as abdominal obesity, hypertension, and insulin resistance. High bilirubin levels can protect metabolic health by reducing oxidative stress and inflammatory responses. Metabolic syndrome includes several components: abdominal obesity, hypertension, high blood glucose, insulin resistance, and dyslipidemia. The inverse relationship between high bilirubin levels and these components suggests that individuals with higher bilirubin levels are less likely to develop metabolic syndrome. Mechanistically, bilirubin reduces insulin resistance through its antioxidant effects, thereby improving metabolic function. Additionally, bilirubin can inhibit chronic inflammation in adipose tissue, preventing abdominal obesity and other components of metabolic syndrome. An epidemiological study involving thousands of participants found that those with higher serum bilirubin levels had a significantly lower risk of developing metabolic syndrome, supporting the protective role of bilirubin in metabolic health (46). 3. Bilirubin and Diabetes: The antioxidant properties of bilirubin may protect pancreatic β-cells from oxidative damage, reducing the risk of diabetes. Pancreatic β-cells are responsible for insulin secretion, and protecting these cells helps prevent diabetes. Studies have shown that high bilirubin levels are associated with a lower incidence of type 2 diabetes. Mechanistically, bilirubin reduces insulin resistance through its antioxidant effects, thereby improving glucose metabolism. Epidemiological studies have found that individuals with higher serum bilirubin levels have a significantly lower incidence of type 2 diabetes, further supporting the role of bilirubin in diabetes prevention. *In vitro* experiments have demonstrated that bilirubin can protect pancreatic β-cells from oxidative stress damage induced by high glucose and high-fat diets. Animal model studies have also confirmed that bilirubin supplementation can improve glucose metabolism and insulin sensitivity, preventing the onset of diabetes. 4. Bilirubin and Cancer: The antiproliferative and pro-apoptotic effects of bilirubin are believed to have protective effects against certain cancers. For example, bilirubin can reduce the occurrence of certain types of cancer by inhibiting cancer cell proliferation and promoting apoptosis. Mechanistically, bilirubin can interfere with cancer cell signaling pathways, inhibiting their proliferation rate, and activating apoptosis-related signaling pathways (e.g., Caspase family), promoting programmed cancer cell death. Some studies have found that individuals with higher serum bilirubin levels have a lower incidence of certain types of cancer, including colorectal, lung, and liver cancers. *In vitro* cell experiments have shown that bilirubin significantly inhibits cancer cell proliferation and induces apoptosis. Animal experiments have also confirmed that bilirubin supplementation can significantly inhibit tumor growth and reduce metastasis, providing strong support for the potential of bilirubin in cancer prevention and treatment (43, 47).

Research on Bilirubin as a Risk Factor for Diseases: 1. High Bilirubin Levels in Liver Diseases: Bilirubin is a metabolic product of the liver, and elevated bilirubin levels usually indicate liver dysfunction. Liver diseases such as hepatitis, cirrhosis, and liver cancer can disrupt bilirubin metabolism, leading to increased serum bilirubin levels. When the liver is damaged, it cannot effectively clear bilirubin from the blood, resulting in its accumulation in the body and causing jaundice. These high bilirubin levels not only indicate liver damage but may also exacerbate pathological changes in the liver. Studies have shown that patients with chronic hepatitis have significantly higher serum bilirubin levels than healthy controls, and these levels correlate positively with the degree of liver fibrosis. 2. Biliary Obstruction and Gallstones: Biliary obstruction (e.g., gallstones, bile duct cancer) can impede bile flow, causing bilirubin to accumulate in the body and resulting in jaundice. Long-term biliary obstruction can lead to bile duct infections, cholangitis, and even bile duct cancer. Patients often present with jaundice, abdominal pain, and fever, with high bilirubin levels being a key diagnostic indicator. One study found that total bilirubin levels in bile duct cancer patients were significantly higher than in patients with cholangitis, and bilirubin levels could predict patient prognosis. 3. Pancreatitis: Patients with acute or chronic pancreatitis often exhibit elevated bilirubin levels due to inflammation-induced bile duct compression or obstruction. High bilirubin levels are important in diagnosing and monitoring pancreatitis. Symptoms of pancreatitis include abdominal pain, nausea, vomiting, and jaundice. Elevated serum bilirubin levels can indicate the severity of pancreatitis. Research shows that serum total bilirubin levels in acute pancreatitis patients are positively correlated with disease severity, suggesting that higher bilirubin levels indicate more severe disease and poorer prognosis (48). 4. Hemolytic Anemia: In hemolytic anemia, accelerated red blood cell destruction leads to elevated serum indirect bilirubin levels. Causes of hemolytic anemia include hereditary spherocytosis, sickle cell anemia, and autoimmune hemolytic anemia. Patients may present with anemia, jaundice, and splenomegaly. High bilirubin levels play an important role in the diagnosis and differential diagnosis of hemolytic anemia. Studies have found that patients with hereditary spherocytosis have significantly elevated indirect bilirubin levels, which correlate positively with the degree of hemolysis. Observational studies have shown that liver function impairment can exacerbate the severity of hyperthyroidism. Researchers analyzing clinical data from a large number of hyperthyroid patients found a positive correlation between liver function impairment and the severity of hyperthyroidism. Liver function impairment affects the metabolism of thyroid hormones, leading to their accumulation in the body and worsening hyperthyroidism. This indicates that liver function status may be a crucial factor influencing the progression and severity of hyperthyroidism. 5. Cushing’s Syndrome: Patients with Cushing’s syndrome, due to prolonged exposure to high cortisol levels, may experience liver function impairment, which affects bilirubin metabolism. These patients may exhibit elevated bilirubin levels along with typical Cushing’s syndrome symptoms such as moon face, central obesity, and purple striae. Studies have shown that elevated serum bilirubin levels in Cushing’s syndrome patients correlate with disease severity and liver function impairment. 6. Hyperthyroidism: The high metabolic state in hyperthyroidism patients may affect bilirubin metabolism and clearance, leading to elevated bilirubin levels. Research shows that increased bilirubin levels in hyperthyroid patients may correlate with disease severity. Elevated bilirubin levels in hyperthyroid patients may present as jaundice, requiring differentiation from other causes of elevated bilirubin. One study indicated that total bilirubin levels in hyperthyroid patients were positively correlated with thyroid hormone levels (e.g., T3 and T4), meaning that higher thyroid hormone levels were associated with higher serum total bilirubin levels. This suggests that bilirubin levels can reflect the severity of hyperthyroidism and serve as an auxiliary indicator for assessing its severity. For suspected hyperthyroidism patients, elevated serum bilirubin levels, especially with other hyperthyroid symptoms, can help support the diagnosis (49, 50).

In summary, bilirubin exhibits dual roles as a protective or risk factor in different disease contexts, with specific effects depending on the disease mechanism and metabolic background.

Based on existing research, the causal relationship between bilirubin and hyperthyroidism has not been clearly elucidated. However, in our study, we provided sufficient evidence of a causal relationship between the two by employing Mendelian randomization combined with meta-analysis. Specifically, we first preprocessed the GWAS data of 35 urinary standard metabolites and performed Mendelian randomization (MR) analysis with hyperthyroidism data from the UKB database and the Thyroidomics Consortium database, respectively. Subsequently, we conducted a meta-analysis of the main results from the two MR analyses using the inverse-variance weighted (IVW) method to ensure the diversity of data sources and achieve triangulation of the study. Finally, to reduce the occurrence of Type I errors, we performed multiple corrections on the significance P-values after the meta-analysis. Ultimately, the evidence showed a significant causal relationship between total bilirubin and hyperthyroidism, with bilirubin being a risk factor for hyperthyroidism. Through reverse MR analysis, we further confirmed that there is no reverse causal relationship between the two.

When total bilirubin levels are elevated, they may affect the development and severity of hyperthyroidism through various mechanisms: 1. Liver Dysfunction: The liver is one of the main organs involved in the metabolism and transport of thyroid hormones. Liver dysfunction may affect the metabolism and transport of thyroid hormones, leading to elevated levels of thyroid hormones in the body. They may worsen hyperthyroidism by reducing the metabolism and clearance rate of thyroid hormones and decreasing the synthesis and function of thyroid hormone transport proteins (51). 2. Binding of Bilirubin to Thyroid Hormones: Bilirubin can form complexes with thyroid hormones, which may affect the metabolism and activity of thyroid hormones in the body. Specifically, bilirubin may bind to thyroid hormones, obstructing their binding to receptors, thereby reducing the biological activity of thyroid hormones, leading to elevated levels of thyroid hormones in the blood and exacerbating the severity of hyperthyroidism (52, 53). 3. Influence of Liver on Thyroid Hormone Transport: The liver plays a role in the body’s transport and clearance of thyroid hormones. When liver function is impaired, it may affect the transport and clearance of thyroid hormones, leading to their accumulation in the body (54, 55). They may worsen hyperthyroidism by reducing the rate of thyroid hormone transport and increasing the plasma half-life of thyroid hormones. 4. Thyroid Hormone Metabolic Pathways: The metabolism of thyroid hormones is regulated by various factors, including enzyme systems in the liver and the synthesis and secretion of bile acids. Accumulation of bilirubin may interfere with the normal functioning of these metabolic pathways, affecting the clearance and breakdown of thyroid hormones and leading to elevated levels of thyroid hormones in the body (56). 5. Immune Regulation: Bilirubin may have immunomodulatory effects on the body, including regulating inflammatory responses and immune cell function. Hyperthyroidism is often associated with activation of the immune system, and increased inflammatory responses and elevated bilirubin levels may further promote the development of hyperthyroidism by affecting immune regulation (57, 58). 6. Oxidative Stress: Bilirubin has antioxidant properties in the body and can scavenge free radicals and alleviate oxidative damage (59). However, when bilirubin levels are excessively high, it may lead to excessive production of oxidative products, exacerbating oxidative stress. Increased oxidative stress may further promote the development of hyperthyroidism (60).

Overall, bilirubin acts as a pathogenic or aggravating factor for hyperthyroidism (hyperthyroidism), and its primary mode of action may be as follows: Firstly, high levels of bilirubin may increase the metabolic burden on the liver, leading to liver dysfunction. The liver is an important organ for thyroid hormone metabolism, and impaired liver function may result in disordered thyroid hormone metabolism, thereby exacerbating hyperthyroidism symptoms. Additionally, hyperthyroidism itself can cause increased cardiac output and changes in liver blood flow, further affecting bilirubin metabolism and clearance, making it difficult for the liver to effectively process the excess bilirubin, leading to elevated levels. Secondly, high bilirubin levels may exacerbate hyperthyroidism by increasing cellular oxidative stress and inflammatory responses. Although bilirubin has antioxidant properties in moderate amounts, at high levels, it may induce oxidative stress, damaging thyroid cells and promoting the excessive release of thyroid hormones, thereby worsening hyperthyroidism symptoms. Simultaneously, elevated bilirubin levels may lead to the release of inflammatory mediators such as TNF-α and IL-6, which can interfere with the normal function of thyroid cells, further promoting the excessive secretion of thyroid hormones. Thirdly, high bilirubin levels may directly act on thyroid cells, affecting their function and hormone synthesis and release through interactions with specific receptors or signaling pathways within the cells, and potentially inducing thyroid cell apoptosis or affecting their proliferation, leading to thyroid dysfunction and promoting the development and aggravation of hyperthyroidism. Lastly, elevated bilirubin levels may interfere with the transport of thyroid hormones in the blood, affecting their binding to transport proteins and resulting in more free thyroid hormones circulating in the blood, exacerbating hyperthyroidism symptoms. Additionally, bilirubin may affect the metabolic processes of thyroid hormones in the liver and other organs, slowing down their clearance and thus increasing their concentration in the body. Overall, these multiple mechanisms of action collectively may lead to the excessive secretion of thyroid hormones and the worsening of hyperthyroidism symptoms, revealing the potential complex role of bilirubin in the pathogenesis of hyperthyroidism.

In summary, elevated total bilirubin levels may affect the development and severity of hyperthyroidism through various mechanisms. These mechanisms interact, collectively influencing the levels and activity of thyroid hormones in the body, thereby affecting hyperthyroidism’s clinical manifestations and progression. Therefore, reducing bilirubin levels is a crucial therapeutic measure when it is elevated in patients with hyperthyroidism, as it may slow the progression of hyperthyroidism and reduce its risk.

The study, through the use of Mendelian randomization combined with meta-analysis, has provided robust evidence for the first time that there is a significant causal relationship between total bilirubin and hyperthyroidism (hyperthyroidism), suggesting that bilirubin may be a pathogenic or aggravating factor for hyperthyroidism. This discovery brings several important prospects. Firstly, it offers a new biomarker for the early diagnosis and risk assessment of hyperthyroidism. By detecting bilirubin levels, doctors can identify high-risk individuals for hyperthyroidism earlier, allowing for early intervention and treatment, which may significantly improve patient outcomes. Secondly, future treatment strategies might consider controlling hyperthyroidism symptoms by regulating bilirubin levels, such as using drugs or methods to reduce serum bilirubin levels. This could provide more treatment options for hyperthyroidism patients and potentially reduce the side effects of traditional therapies. Additionally, this finding prompts further research into the specific role of bilirubin in the pathogenesis of hyperthyroidism. Understanding how bilirubin exacerbates hyperthyroidism by affecting liver function, thyroid hormone metabolism, immune responses, and oxidative stress will help deepen our understanding of the disease’s mechanisms and lead to the development of more targeted treatments. Lastly, this research also highlights the need to consider other diseases or factors that may cause elevated bilirubin levels in hyperthyroidism patients. Preventing and treating conditions associated with increased bilirubin levels could be important in reducing the incidence and severity of hyperthyroidism, thereby improving overall patient health.

In conclusion, the study opens new avenues for the diagnosis and treatment of hyperthyroidism. By thoroughly investigating the mechanisms of bilirubin’s role, it is hoped that more effective and safer treatment strategies can be developed in the future, significantly enhancing the quality of life for hyperthyroidism patients.

MR studies have explored the relationship between bilirubin and hyperthyroidism at the genetic level and, based on this, conducted meta-analyses, providing a more precise understanding of the connection between the two. This research has more accurately elucidated the causal relationship between the two based on observational studies. However, this study still has limitations, mainly due to data acquisition limitations, as the sample is limited to European populations. Therefore, the results may only partially represent some populations globally.

## 6 Conclusions

Through Mendelian randomization (MR) combined with meta-analysis, the study found a positive correlation between total bilirubin levels and hyperthyroidism, indicating that total bilirubin is a risk factor for hyperthyroidism and may accelerate the onset and progression of the disease. This finding has significant practical implications. Firstly, it provides us with a deeper understanding, enabling better identification of individuals at risk for hyperthyroidism and the implementation of preventive measures. By early detection of total bilirubin levels, clinicians can identify high-risk groups and intervene early, thereby significantly improving outcomes.

Moreover, monitoring and controlling total bilirubin levels could be an effective treatment strategy in managing hyperthyroidism. By regulating total bilirubin levels, we can reduce unnecessary harm to patients and provide more precise treatment, thus improving their quality of life and treatment outcomes. Monitoring and regulating total bilirubin levels can help manage hyperthyroidism and reduce the occurrence of complications. This research provides new insights and directions for the management and treatment of hyperthyroidism, holding significant clinical importance. It advances clinical practice, making treatment more personalized and effective.

## Data Availability

The original contributions presented in the study are included in the article/**Supplementary Material**. Further inquiries can be directed to the corresponding author.

## Supplementary material

Supplementary Table 1 Calculate the F-statistic for Each SNP.

Supplementary Table 2 Results of the pleiotropy test.

Supplementary Table 3 Results of the heterogeneity test.

Supplementary Table 4 Results of MR analysis.

Supplementary Table 5 Results of the meta-analysis.

Supplementary Table 6 Results after multiple corrections.

Supplementary Table 7 Results of reverse validation of positive indicators.
